# Interfacial and Transport Phenomena between Liquid Metal and Solid Structural Materials

**DOI:** 10.3390/ma15196851

**Published:** 2022-10-02

**Authors:** Donatella Giuranno, Wojciech Polkowski

**Affiliations:** 1 National Research Council (CNR-ICMATE), Via de Marini, 6, 16149 Genoa, Italy; 2 Łukasiewicz Research Network—Krakow Institute of Technology, Zakopiańska 73 Str., 30-418 Kraków, Poland

## 1. Introduction

Research activities in materials science typically range from basic and curiosity-motivated research to that which is applications-oriented, and the well-known Materials Science paradigm is usually followed: Processing → Microstructure → Properties → Performance.

Nowadays, thanks to the use of computational models, an inversed trend is observed, and the Material Designs approach is successfully taking off. Material design basically focuses on the design and production of the new materials that exhibit novel tailored structures and properties that meet the requirements of specific applications. In order to succeed, the links that these materials have with the basic research cannot be disregarded. In particular, taking into account the influence of the process/working conditions on the final developed microstructures, the investigations that are carried out by following the systematic approach of basic research still remain crucial. Such comprehensive and multidisciplinary investigations enable us to optimize the manufacturing of advanced materials via vapour-, solid- and liquid-assisted processes such as PVD and CVD, solid-state reactions, casting and solidification, metal infiltration, etc. In addition, the post-production phenomena that occur during the thermal treatments or those that are induced upon the exposure of the material to high temperatures and ag-gressive environments, can be predicted. In particular, the interaction phenomena that occur between dissimilar materials such as metals and ceramics are of particular relevance. 

The aim of this Special Issue was to stimulate researchers worldwide to share their most interesting experiences and know-how on the interaction that are observed when liquid metals are in contact with solid materials. Finally, the Special Issue is a collection of five original research articles and one review paper that describe current research trends and future perspectives in the manufacture of tailored, advanced materials for highly demanding applications. Short descriptions of each paper that is included in our Special Issue are given below.

The review paper by Li et al. [1] summarizes the recent advances in the development of Cu diffusion-proof materials, including metals, metal alloys, self-assembled molecular layers (SAMs), two-dimensional (2D) materials and high-entropy alloys (HEAs). The authors have also highlighted the challenges in this and the future research directions.

Kim et al. [2] have used the CALPHAD (CALculation of PHAse Diagram) to investigate the phase diagram of an arbitrary A–B nanoparticle system that is placed under increased pressure. The authors found that the eutectic temperature decreased in most of the cases, except when the interaction parameter in the liquid was zero and when the solid was positive while the excess volume parameter of the liquid was also positive. Under these conditions, the eutectic temperature increased with increasing pressure.

The paper that is presented by Dybalska et al. [3] discusses the role of a contactless melt sonicating treatment in the enhancement of the mechanical properties of pure Al. The authors demonstrated that there was a positive effect when introducing ultrasound waves into liquid metal on the quality of the casting and the resultant mechanical properties. When it was compared to an untreated material, pure aluminum that was produced under the proposed treatment showed an increase in both the strength and ductility. The main mechanism that is behind this improvement in mechanical properties was associated with a ultrasonically induced grain refinement effect.

The next paper described the result of the experimental studies that were conducted on practical implementation of the high-temperature capillarity phenomena, namely, ones involving a reactive wetting and reactive melt infiltration. In this topic, Zhang et al. [4] described pioneering findings on the interaction between Sn-V active solders and porous graphite at a temperature range of 650–950 °C. It was shown that even a very small addition of vanadium (0.5 wt %) to tin strongly improved the wetting, while a further increase of its content (up to 7 wt %) only slightly affected the contact angle values. The spreading kinetic values of the Sn-V alloys on porous graphite well fit the classic product reaction-controlled model. This work is believed to provide implicative notions on the fabrication of graphite-related materials and devices using novel V-containing bonding alloys.

Work that was conducted by Novakovic et al. [5] is a case study on designing composites by employing a melt infiltration process by using liquid Ir-Si Alloy/SiC systems as an example. The authors provide original data on the Gibbs free energy of the mixing of the Ir-Si liquid phase which was calculated using Miedema’s model and the free volume theory. Subsequently, the surface properties were calculated using the quasi-chemical approximation (QCA) for the regular solution, while to obtain the viscosity, the Moelwyn-Hughes (MH) and Terzieff models were applied. Based on the above-mentioned data, the parabolic Washburn’s equation was used to design the non-reactive infiltration of the Ir-Si/SiC systems in terms of their infiltration depth during the process for different temperatures, times, and pore sizes of the SiC preforms.

Finally, Giuranno et al. [6] performed sessile drop-based experiments at 1450 °C in order to evaluate the course of the interaction between the molten silicon-rich Si-Ti alloys (8, 16.2 and 24 at % Ti) and the glassy carbon. It has been documented that a very good wetting process promotes a reactive infiltration that evolves according to a spontaneous mechanism, while SiC has been recognized as the only one interfacial product. Thus, a Si-Ti system has been recommended as an excellent candidate for infiltrating the metal materials for C- and SiC-based porous systems to fabricate the SiC/TiSi_2_ ultra-high temperature composites. 

We hope that this Special Issue will be interesting for the scientific and academic communities, and it will bring a lot of inspiration for future research activities in the field of designing of advanced materials and their fabrication processes. 

## Data Availability

Not applicable.
